# Epidemiological studies of sleep disorder in educational community of Pakistani population, its major risk factors and associated diseases

**DOI:** 10.1371/journal.pone.0266739

**Published:** 2022-04-21

**Authors:** Ali Umar, Muhammad Saleem Khan, Sheikh Arslan Sehgal, Kamran Jafar, Shabbir Ahmad, Ahmad Waheed, Muhammad Waseem Aslam, Muhammad Wajid, Tanzil Ur Rehman, Tehmina Khan, Allah Ditta, Hasnain Akmal, Muhammad Ashfaq, Tariq Javed, Rida Tahir

**Affiliations:** 1 Department of Zoology, Faculty of Life Sciences, University of Okara, Okara, Pakistan; 2 Department of Bioinformatics, Faculty of Life Sciences, University of Okara, Okara, Pakistan; Sapienza University of Rome, ITALY

## Abstract

Sleep is one of the most important functions of the life. The disturbance in sleep or quality of sleep leads to several dysfunctions of the human body. This study aimed to investigate the prevalence of sleep disorders, their possible risk factors and their association with other health problems. The data was collected from the educational community of the Pakistani population. The Insomnia Severity Index (ISI) was used to evaluate the insomnia and the sleep apnea was evaluated through a simple questionnaire method. The blood samples were collected to perform significant blood tests for clinical investigations. Current research revealed that the individuals in the educational community had poor sleep quality. A total of 1998 individuals from the educational community were surveyed, 1584 (79.28%) of whom had a sleep disorders, including insomnia (45.20%) and sleep apnea (34.08%). The measured onset of age for males and females was 30.35 years and 31.07 years respectively. The Clinical investigations showed that the sleep had significant impact on the hematology of the patients. Higher levels of serum uric acid and blood sugar were recorded with a sleep disorder. The individuals of the educational community were using the sleeping pills. The other associated diseases were mild tension, headaches, migraines, depression, diabetes, obesity, and myopia. The use of beverage, bad mood, medical condition, mental stress, disturbed circadian rhythms, workload and extra use of smartphone were major risk factors of sleep disorders. It was concluded that the insomnia was more prevalent than the sleep apnea. Furthermore, life changes events were directly linked with disturbance of sleep. Tension, depression, headaches, and migraine were more associated with sleep disorders than all other health issues.

## Introduction

Sleep is one of the most important functions of life. Among *Homo sapiens*, an individual usually sleeps for one-third of one’s life. According to National Sleep Foundation, 12–15 h/night for Kids (4–11 months), 11–14 h/night for kids (1–2 years), 9–11 h/night for children (6–13 years), 8–10 h/night for teenagers (14–17 years), and 7–8 h/night sleep is recommended for adults [1]. The core centre, hypothalamus controls the sleep through circadian rhythms. Circadian rhythms (24 hours clock) are regulated by external factors and time cues. Rapid eye movement (REM) and non-rapid eye movement (non-REM) are types of sleep recorded in humans, occurs in a cyclic process. About 75–80% of sleep cycles comprise non-REM while the remaining 20–25% is REM sleep. At the beginning of sleep, four non-REM sleep cycles occur progressing to REM sleep cycles. There are 4–6 non-REM and REM sleep cycles in a night, each lasting for 70–120 minutes [2, 3].

Sometimes an individual may sleep less time as recommended by World Health Organization (WHO) [4]. The main cause of less sleep could be the disturbance in normal sleep pattern. The disturbed sleep is a critical issue of the current era around the globe. The young and older adults of both the genders are suffering equally from different kinds of sleep disorders and reported in 50% of the oldsters (≥ 60 years) that cannot enjoy a peaceful sleep. Lifestyle, medical conditions, long-time use of medicines, life changes events such as work stress, spousal death, retirement and age are major factors that influence the sleep patterns greatly [5]. Although, OSAs are equally dispersed by age in the overall population and manifest differently in adults and children. The adults are the most likely to be obese, with an excess of adipose tissue, necessitating a series of therapies to minimize the disease. In youngsters, adenotonsillar hypertrophy is the primary cause, with up to 90% of the cases responding well to adenotonsillectomy [6]. The sleep patterns are also changed significantly with the aging process. Additionally, overuse of smartphones disturbs the sleep-wake cycle leading to sleep disorder. Furthermore, Pasquali, Colella [7] related the sleep apnea and treatment of obesity. It has been evidenced that the weight loss sleep quality was improved in patients.

People with disturbed sleep cannot perform well and may suffer from various other health complications. It may also initiate the pathological conditions in the human body that disturb the cortical functions, metabolic functions, and endocrine functions [8, 9]. Neurological malfunctions are also linked with disturbance in sleep [10]. The obstructive sleep apnea has lately been linked to chronic inflammatory nasal disorders such as allergic rhinitis and vasomotor rhinitis, revealing a clear link between nasal problems and the sleep severity [11]. The chronic hypoxia and sleep fragmentation can lead to nervous system diseases, such as impaired neurocognitive function and memory loss [12].

Previous study revealed that the disturbance in sleep patterns enhances the morbidity rate, death rate, physical functioning, and change in life quality in the older population [13]. The sleep duration decreases as age increases, as previously studied that the older people take more time to fall asleep, frequently wake up at night, and get up very early in the morning [14, 15].

One of the primary sleep problems is sleep insomnia. Insomnia is a condition in which an individual cannot fall asleep or stay asleep [16, 17]. This sleep problem affects deep sleep patterns badly. Insomnia is a short-term disorder that lasts from a few days to a chronic condition lasting for many years. The psychological problem, diseases, long-term use of drugs, and behavioural problems due to environmental changes are key factors for insomnia in older age of ≥ 60 years [13]. During the survey of the National Sleep Foundation, it was reported that the increased sleep insomnia in older populations was positively associated with co-morbid medical conditions such as depression, respiratory and cardiac issues [18]. Furthermore, in terms of diagnosis of sleep disorders (obstructive sleep apnea), the STOP-BANG Questionnaire has proven to be a more accurate tool for detecting mild, moderate and severe conditions [19]. The sleep disorders are neglected health issues and most of the population is suffering from one or more kind of sleep disorder without knowing the consequences of these health issues. Extensive literature review was performed and it was observed that no study has been performed against age of onset in older individuals. In present study, age of onset in older individuals for sleep disorder along with prevalence, risk factors and associated diseases of sleep disorders were critically analysed in an educational community.

## Materials and methods

### Study population

**Fig 1:**
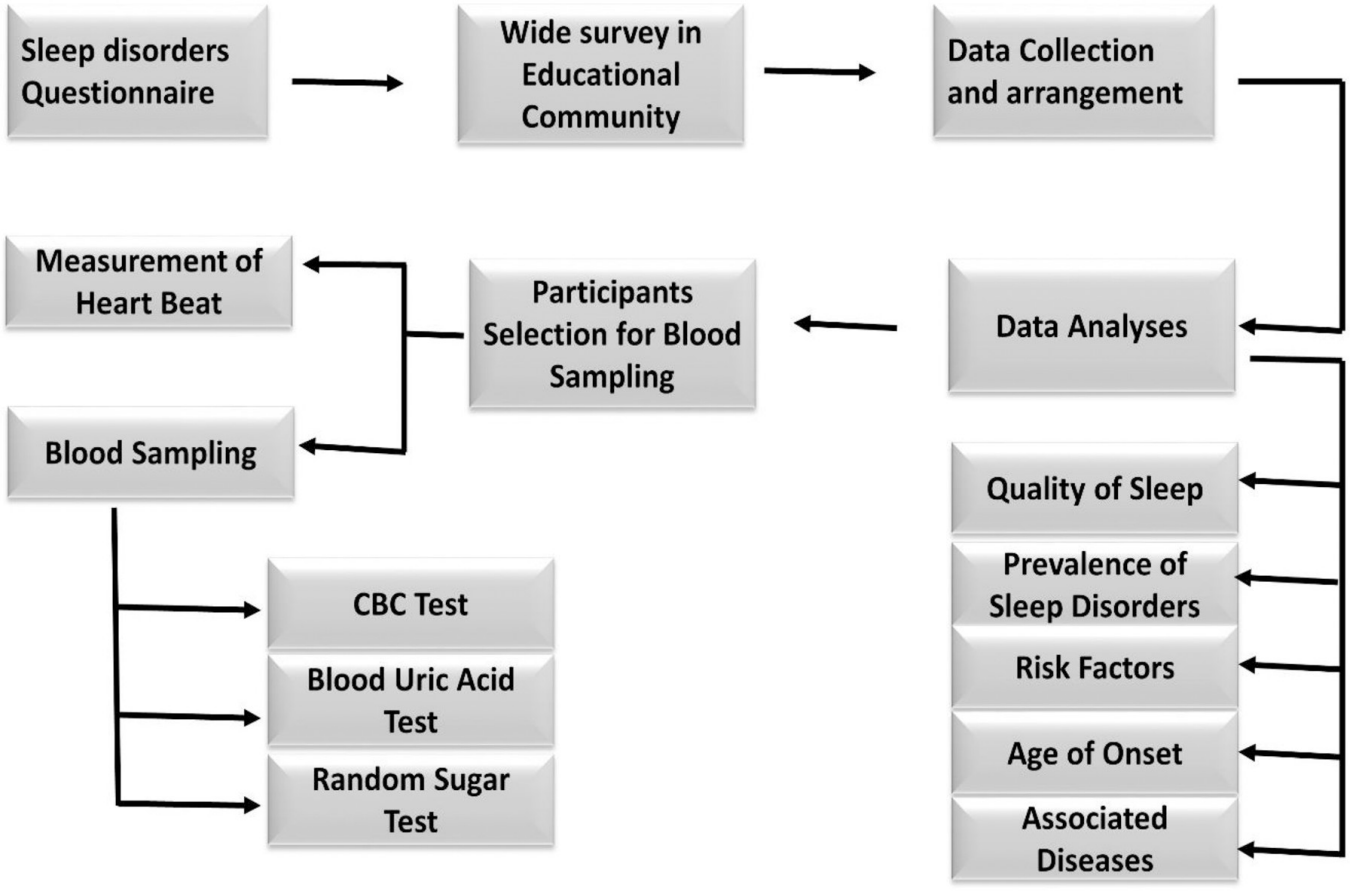
Flow sheet of research methodology.

The study was conducted on the educational community in both the public and private sectors schools, colleges, and universities in the province of Punjab, Pakistan. Central Survey and Home Survey have adopted differing approaches for data collection and data analyses. Every participant included in this survey studies was interviewed by the investigator lead to fill out self-questionnaires (S1 and S3 Files). The teaching staff was selected for this study. Other than teaching staff, non-teaching staff and students were barred from participating in the study (Fig 1).

### Data collection

#### General survey for sleep disorder patients

To assess sleep patterns, the prevalence of sleep disorders, and its associated health problems such as obesity, migraine, inflammation, infection, and the effect of sleep disorders on life of respondents within the educational community, including school education and higher education. A wide survey among the educated population in the province of Punjab, Pakistan was conducted. The survey was done through a questionnaire. Different questions related to sleep, physical activity, lifestyle, and mood swing was also considered for the participants and responses were observed.

### Insomnia Severity Index (ISI)

Insomnia is the most common complaint however remains largely an unrecognized public health problem. The Insomnia Severity Index (ISI) was used to find the prevalence of insomnia among the educated population. The items evaluate the severity of sleep difficulties, maintaining sleep and early morning awakening, the degree of satisfaction with current sleep patterns, interference in daily operation, perceptible sleep impairment by others, and sleep difficulties. Each item has a 5-point Likert scale, with an overall score between 0–28. The following were interpreted: no clinical insomnia (0–7), insomnia sub threshold (8–14), mild insomnia (15–21), and severe insomnia (22–28). Initially, a cut-off score of 15 was proposed for clinical insomnia [20].

## Sleep apnea evaluation

Sleep apnea was evaluated through detailed questionnaire method [21] (S1 and S3 Files).

### Clinical investigations

After assessing sleep patterns and recognizing people with sleep disorders, further assessment of associated health problems was done among the selected participants. The blood samples were collected to perform a blood tests and medical tests (complete blood count CBC, blood uric acid test, blood sugar level, and heartbeat per minute) to assess associated diseases with the sleep disorder (S2 File).

### Statistical analyses

The Minitab (version 19.0) software was utilized to analyze data from a complex sampling design. All calculations were weighted by variables (gender, age groups, and qualifications) based on population data from the study area. The analyses included all participants with available interest variables and missing data were not used. The Rao Scott *Chi-Square* Test compared one symptom of sleep apnea and insomnia to two symptoms, three symptoms, and four symptoms of sleep apnea and insomnia. In terms of data, a *P* value of less than 0.05 was considered significant [22].

### Study ethics

All the participants were told about the procedures and study criteria. Verbal consent was taken from those who participated in the study, in order to avoid any type of inconvenience in the future. Written consent from the participants was waived by the Ethics Committee Department of Zoology, University of Okara, Okara Pakistan (S4 File).

## Results

This study covers the prevalence rate of sleep disorders including insomnia and sleep apnea along with their risk factors and their association with other diseases among the educational community. The study involved male and female teachers, lecturers, and professors from private and public sectors schools, colleges, and universities.

### Respondents demography

The 2540 people provided the consent and the data was selected for 1998 participants. Among 1998 participants, 990 (49.55%) were male and 1008 (50.45%) were female participants.

The age of the respondents ranged between 22 to 60 years, with the highest numbers falling in the 26–35 years of age group (n = 714, 35.74%). The second-highest numbers of respondents were in the age group of ≤ 25 years (n = 621, 31.08%), followed by 36–45 years (n = 396, 19.82%), while respondents from the age group of >45 years (n = 267, 13.29%) were low. Thus, the majority of the respondents (82.5%) were in the mature adult and young-adult age groups (26–35 years & ≤ 25 years), while the older adults (>45 years) were few (13.29%). More of the respondents were from government schools among all age groups (Table 1).

**Table 1:** Demographic characteristics of the respondent.

Participant Characteristics	Respondents	
	N	%
**Gender**		
**Male**	990	49.55
**Female**	1008	50.45
**Age**		
**≤ 25 years**	621	31.08
**26–35 years**	714	35.74
**36–45 Years**	396	19.82
**>45 Years**	267	13.36
**Qualifications**		
**Graduation**	1437	71.92
**Post-Graduation**	561	28.08
**Sector**		
**Govt. Teachers**	975	48.8
**Private Teachers**	1023	51.2

### Sleep duration and sleep quality

From Table 2, it was clear that gender has significant impacts on duration and quality of sleep as the *p-*value was observed 0.03. A high significant level (*p-*value = 0.00) of the age factor for the quality of sleep was also observed in this study. More sleepers were found in all respondents than long sleepers and the sector (*p-*value = 0.005) where respondents were working and the level of their qualifications (*p-*value = 0.00) were also positively linked with the sleep durations.

**Table 2:** Sleep durations of respondents.

Participant Characteristics	Shorter Sleepers (N = 837)		Normative Sleepers (N = 876)		Longer Sleepers (N = 285)		X^2^	P-Value
	N	%	N	%	N	%		
**Gender**								
**Male**	444	53.05	411	46.92	135	47.37	7.06	0.03**
**Female**	393	46.95	465	53.08	150	52.63		
**Age Groups**								
**≤ 25 years**	282	33.69	267	30.48	72	25.26	23.12	<0.001**
**26–35 Years**	258	30.82	351	40.07	105	36.84		
**36–45 Years**	183	21.86	150	17.12	63	22.11		
**>45 Years**	114	13.62	108	12.33	45	15.79		
**Sector **								
**Private**	417	49.82	441	50.34	165	57.89	6.01	0.05*
**Government**	420	50.18	435	49.66	120	42.11		
**Qualifications**								
**Graduation**	576	68.82	633	72.26	228	80	13.25	<0.001**
**Post-Graduation**	261	31.18	243	27.74	57	20		

** = Highly significant (P<0.01) NS = Non-significant (P>0.05)

* = Significant (P<0.05), X^2^ = Chi-square.

#### Prevalence of sleep disorders

In this study, it was observed that the prevalence rate of two common types of sleep disorders insomnia and sleep apnea among the individuals of the educational community. A total of 1998 individuals from the educational community were surveyed, 1584 (79.28%) of whom had a sleep disorders, including insomnia (45.20%) and sleep apnea (34.08%). Among both genders prevalence rate was higher in females (41.14%) as compared to males (38.14%) while among age groups individuals higher rate was observed in the age group of 26–35 years (25.53%) followed by ≤ 25 years (24.02%), 36–45 years (15.92%) and then >45 Years (13.81%) (Table 3).

**Table 3:** Prevalence of sleep disorders.

Participant Characteristics	Sleep apnea 34.08%		Insomnia 45.20%		Over all prevalence 79.28%		X^2^	P-Value
	N	%	n	%	n	%		
**Gender**								
**Male**	330	33.33	432	43.64	762	38.14	0.059	0.808
**Female**	351	34.82	471	46.73	822	41.14		
**Age**								
**≤ 25 years**	195	31.4	285	45.89	480	24.02	5.608	0.469
**26–35 Years**	222	31.09	288	40.34	510	25.53		
**36–45 Years**	132	33.33	186	46.97	318	15.92		
**>45 Years**	132	49.44	144	53.93	276	13.81		
**Sector**								
**Private**	360	25.05	450	31.32	810	40.54	1.99	0.369
**Government**	321	57.22	453	80.75	774	38.74		
**Qualification**								
**Graduation**	486	49.85	612	62.77	1098	54.95	3.33	0.189
**Post-Graduation**	195	19.06	291	28.45	486	24.32		

** = Highly significant (P<0.01) NS = Non-significant (P>0.05)

* = Significant (P<0.05), X^2^ = Chi-square.

#### Prevalence of insomnia

Table 4 showed that with a *p-*value of 0.21, gender had no significant impact on the severity and prevalence of insomnia. In this study, the age factor had a high level of significance (p-value = 0.001) for the severity and prevalence of insomnia. Furthermore, the sector in which respondents worked (p-value = 0.16) had no significant impact on the severity and prevalence of insomnia and the level of their qualification (p-value = 0.001) was also positively linked with insomnia.

**Table 4:** Symptoms and prevalence of insomnia.

Participant Characteristics	Insomnia sub threshold 1 symptom (n = 552)		Mild insomnia 2 symptoms (n = 543)		Severe insomnia 3 symptoms (n = 903)		X^2^	P- Value
	N	%	N	%	n	%		
**Gender**								
**Male**	279	28.18	279	28.18	432	43.64	3.109	0.211
**Female**	273	27.08	264	26.19	471	46.73		
**Age **								
**≤ 25 years**	174	28.02	162	26.09	285	45.89	32.992	0.001**
**26–35 Years**	240	33.61	186	26.05	288	40.34		
**36–45 Years**	84	21.21	126	31.82	186	46.97		
**>45 Years**	54	20.22	69	25.84	144	53.93		
**Sector **								
**Private**	276	26.98	297	29.03	450	43.99	3.649	0.161
**Government**	276	28.31	246	25.23	453	46.46		
**Education **								
**Graduation**	405	28.18	420	29.23	612	42.59	16.183	0.001**
**Post-Graduation**	147	27.37	123	22.91	291	54.19		

** = Highly significant (P<0.01) NS = Non-significant (P>0.05)

* = Significant (P<0.05), X^2^ = Chi-square.

#### Prevalence of sleep apnea

Table 5 revealed that gender had no significant impact on the severity and prevalence of sleep apnea as the p-value is 0.59. A highly significant (p-value = 0.001) age factor for the severity and prevalence of sleep apnea was also observed in this study. Furthermore, the sector (p-value = 0.206) where respondents were working suggested a non-significant impact on the severity and prevalence of sleep apnea, and the level of their qualifications (p-value = 0.001) were also positively linked with sleep apnea.

**Table 5:** Symptoms and prevalence of sleep apnea in studied population.

Participant Characteristics	1 symptom (n = 693)		2 symptoms (n = 624)		3 symptoms (n = 681)		X^2^	P-value
	N	%	N	%	n	%		
**Gender**								
**Male**	354	51.08	306	49.04	330	48.46	1.041	0.594
**Female**	339	48.92	318	50.96	351	51.54		
**Age **								
**≤ 25 years**	219	35.27	207	33.33	195	31.40	40.15	0.001**
**26–35 Years**	267	37.39	225	31.51	222	31.09		
**36–45 Years**	150	37.88	114	28.79	132	33.33		
**>45 Years**	57	21.35	78	29.21	132	49.44		
**Sector **								
**Private**	336	32.84	327	31.96	360	35.19	3.161	0.206
**Government**	357	36.62	297	30.46	321	32.92		
**Education **								
**Graduation**	468	32.57	483	33.61	486	33.82	16.002	0.001**
**Post-Graduation**	225	40.11	141	25.13	195	40.11		

** = Highly significant (P<0.01) NS = Non-significant (P>0.05)

* = Significant (P<0.05), X^2^ = Chi-square.

#### Prevalence of sleep disorder and tension

From Table 6, it is clear that gender had no significant impact on the occurrence of tension as the p-value is 0.132. A highly significant (p-value = 0.001) age factor for the occurrence of tension was also observed in this study. Furthermore, the sector (p-value = 0.001) where respondents were working suggested a highly significant impact on the occurrence of tension. The qualification level of respondents (p-value = 0.242) was not positively linked with the occurrence of tension.

**Table 6:** Respondents suffering from tension.

Participant Characteristics	Participants suffering from Tension (N = 1998)					
	Yes (n = 729)	%	No (n = 1269)	%	X^2^	P-Value
Gender						
**Male**	345	34.85	645	65.15	2.272	0.132
**Female**	384	38.10	624	61.90		
**Age Group**						
**≤ 25 years**	210	33.82	411	66.18	45.963	0.001
**26–35 Years**	237	33.19	477	66.81		
**36–45 Years**	135	34.09	261	65.91		
**>45 Years**	147	55.06	120	44.94		
**Sector**						
**Private**	333	32.55	690	67.45	14.009	0.001
**Government**	396	40.62	579	59.38		
**Qualification**						
**Graduation**	513	35.70	924	64.30	1.368	0.242
**Post-Graduation**	216	38.50	345	61.50		

** = Highly significant (P<0.01) NS = Non-significant (P>0.05)

* = Significant (P<0.05), X^2^ = Chi-square.

### Participants using sleeping pills

Table 7 represented the comparison between respondents who were using sleeping pills to attain proper sleep and those who were not using sleeping pills by socio-demographic characteristics. Respondents were divided into three categories: “Yes” (daily use), “Really” (sometimes use of sleeping pills), and “No” (no use of sleeping pills).

**Table 7:** Participants using sleeping pills.

Participant Characteristics	Yes (n = 168)		Rarely (n = 207)		No (n = 1623)		X^2^	P- Value
	N	%	n	%	n	%		
**Gender**								
**Male**	81	8.18	87	8.79	822	83.03	5.585	0.061
**Female**	87	8.63	120	11.90	801	79.46		
**Age Group**								
**≤ 25 years**	45	7.25	57	9.18	519	83.57	47.298	0.001**
**26–35 Years**	69	9.66	75	10.50	570	79.83		
**36–45 Years**	27	6.82	21	5.30	348	87.88		
**>45 Years**	27	10.11	54	20.22	186	69.66		
**Sector**								
**Private**	78	7.62	126	12.32	819	80.06	9.631	0.008**
**Government**	90	9.23	81	8.31	804	82.46		
**Qualification**								
**Graduation**	120	8.35	144	10.02	1173	81.63	0.689	0.712
**Post-Graduation**	48	8.56	63	11.23	450	80.21		

** = Highly significant (P<0.01) NS = Non-significant (P>0.05)

* = Significant (P<0.05), X^2^chi-square.

A high significance (p-value = 0.00) of the age factor for using the sleeping pills was observed in this study. The use of sleeping pills was enhanced with the increase of age so more use of sleeping pills was observed in older adults of the educational community. Furthermore, the sector (p-value = 0.005) where respondents were working was also positively linked with the use of sleeping pills.

### Clinical investigation

Three clinical tests including complete blood count (CBC), Blood Glucose random and serum uric acid were performed to check the impacts of sleep disorder on blood profile, blood glucose profile measurement and uric acid level. The heartbeat of patients was also measured. A Slightly elevated level of WBCs was recorded in sleep disturbed people. The reference value of WBCs was 7.58–15.4x10^3^/μL while obtained value minimum and maximum were 7.58–15.4x10^3^/μL. RBCs were in a normal range. Hemoglobin level (7.1–17 g/dl) was different from lower reference range however from upper reference rage normal. HTC ranged between 22.2–47.5%. Increased red blood cell width distribution (RDW) (11.2–18.4) was obtained from the CBC test. Similarly, increased platelet count (109–509 x10^3^/ μL) was obtained from the CBC test. Blood sugar level was also slightly increased ranging from 76–173. Blood uric acid values ranged between 2.9–8 mg/dl. Heartbeat level was measured up to 122 hr/m (Table 8).

**Table 8:** Clinical assessment with respect to sleep disorder.

Variable	Reference Value	Mean	SD	Range (Minimum-Maximum)
**WBC (x10** ^**3**^ **/μL)**	3.5–10	11.58	2.7	7.58–15.4
**RBC (x10** ^**6**^ **/ μL)**	3.8–5.8	4.86	0.58	3.8–5.45
**HGB (g/dl)**	12.0–17.0	13	2.87	7.1–17
**HCT (%)**	34–47	38.37	8.26	22.2–47.5
**MCV (FL)**	75–95	78.92	11.77	58.1–92.4
**MCH (pg)**	24–32	26.44	4.65	8.6–32
**RDW**	11.0–14.0	14.93	3.61	11.2–18.4
**MCHC (g/dl)**	31–35	32.43	5.02	22.5–36.7
**PLT (x10** ^**3**^ **/ μL)**	150–450	291	122.27	109–509
**Neutrophils**	1.8–7.5	6.4	1.41	4.8–9.9
**Lymphocytes**	1.5–4	3.71	1.01	2.3–5.43
**Monocytes**	0.2–0.8	4.83	2.29	0.94–8
**Eosinophils**	0–0.6	1.75	1.58	0.05–5.9
**Blood Sugar level**	70–130	122.5	32.32	76–173
**Blood Uric acid**	2.4–6	5.73	1.6	2.9–8
**Heart Beat**	60–100	102.70	11.63	87–122

### Associated risk factors

The use of beverages (coffee, tea) is very common in Pakistan, in this study about 54% of respondents were using tea or coffee to get relax. In 32% of respondents disturbed sleep was associated with a medical condition. Workload led to mental stress and interrupted circadian rhythms. Extra use of smartphone is one of the most common risk factors of disturbed sleep wake cycle leading to sleep disorders (insomnia or sleep apnea), about70% of respondents were reported extra use of smart cell phones and were experiencing interrupted sleep wake cycle (Table 9).

**Table 9:** Associated risk factors of sleep disorder (N = 1998).

Risk factor	N	Percentage
**Use of Beverage**	1095	54.80
**Bad Mood**	1146	57.36
**Medical Condition**	645	32.28
**Mental Stress**	729	36.49
**Work load**	588	29.43
**Extra use of smart phone**	1400	70.07

### Association with other diseases

**Fig 2:**
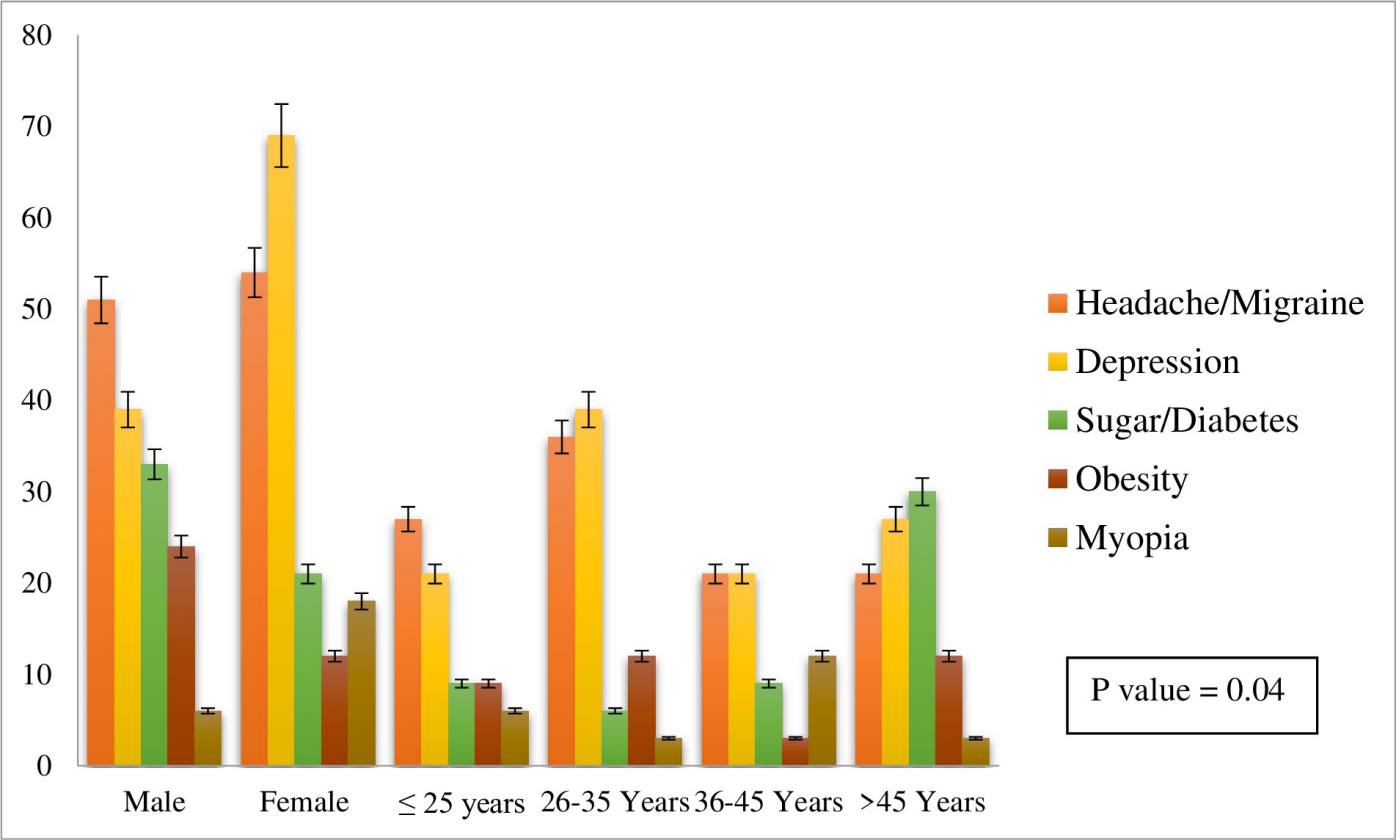
Associated diseases with sleep disorders.

Fig 2 showed that sleep disorders either insomnia or sleep apnea was associated with many other health issues and diseases. Sleep disorders were like Headaches, Migraines, Depression, Sugar or Diabetes, Obesity, and Myopia (Fig 2). These diseases were found in both genders and all other demographic variables. Tension, depression, headaches, and migraine were more prominent with sleep disorders than all other health issues.

### Age onset patterns

**Fig 3:**
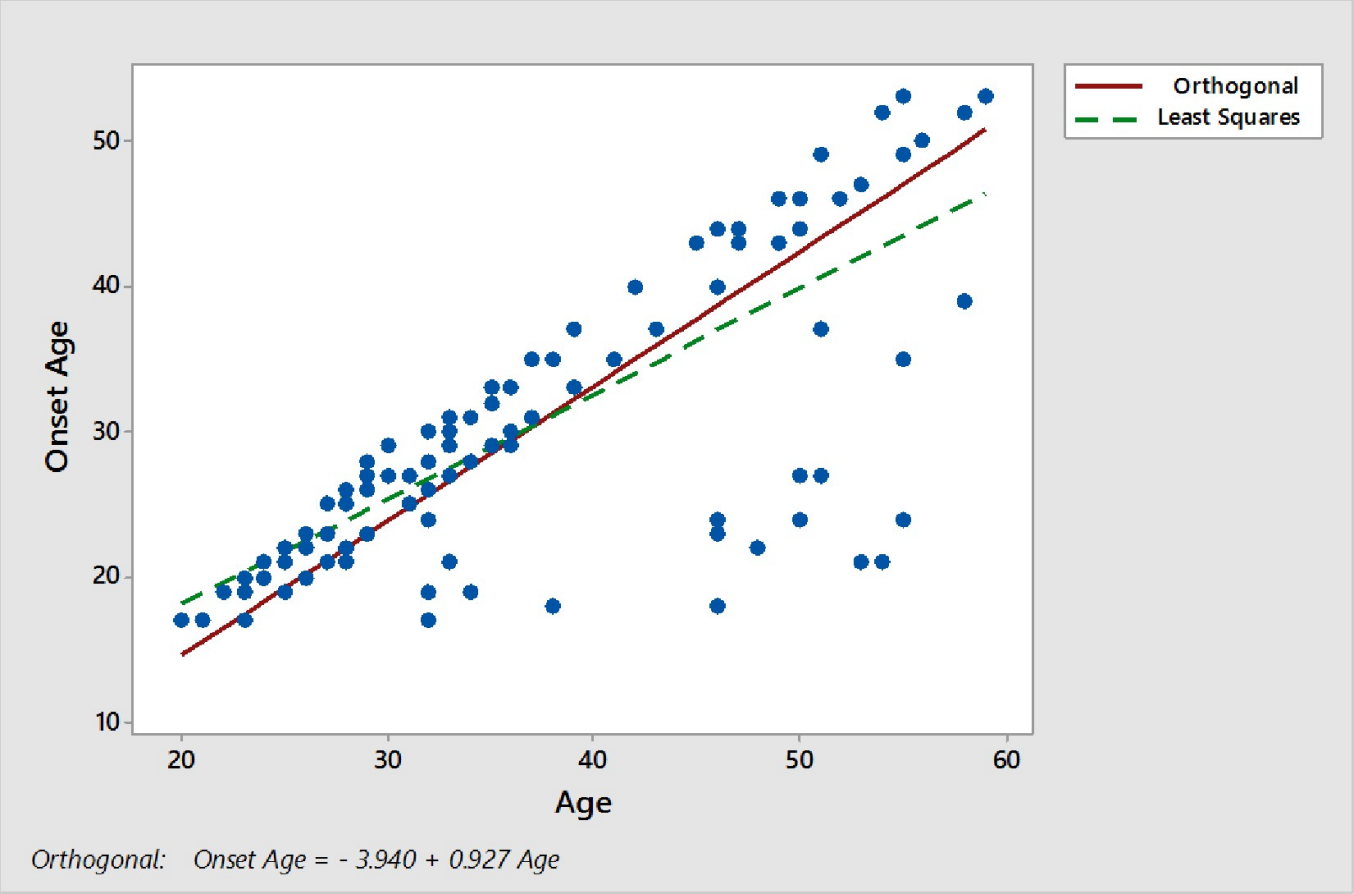
Age of onset of sleep disorders.

Furthermore, we measured the age of onset patterns in sleep disorders in both sexes. Respondents from both genders have the age range of 22 years to 60 years (Fig 3). Not all the respondents had the same age of sleep disorder. Different respondents were suffered from any kind of sleep disorder at different age stages. Onset age patterns obtained from the regression equation is:

Onsetage=−394+0.927(age)

From this equation age onset patterns for male and female gender were computed. There is a slightly difference in onset patterns of age between both genders. The onset of age was measured in males (30.35 Years) and females (31.07 Years). Fig 2 indicate the age onset patterns of sleep disorder either sleep apnea or insomnia.

## Discussion

People having sleep disorders cannot perform effectively and may suffer from a range of other health problems. It may also trigger pathological diseases in the human body that disrupt cerebral, metabolic, and endocrine functioning [8, 9]. The present study highlighted that there was a higher risk of short sleep duration similarly by Liang, Qu [23] that has shown a lower risk of sleeping for 7 hours per night and a higher risk for short sleep duration and long sleep duration Although the link between obstructive sleep apnea and cardiovascular disorders has been established, the role of obstructive sleep apnea treatment in terms of cardiovascular outcomes is debatable, with different treatment options available [24]. Fan, Xu [25] also reported a higher risk of poor sleep quality with a short sleeping duration which is similar to the findings of the present study.

Among all respondents, 45.20% (n = 903) of respondents were at moderate to severe levels of insomnia. A higher prevalence rate of insomnia (45.20%) was observed in the present study than the prevalence of insomnia (19.3%) reported in university students by Albasheer, Al Bahhawi [26] from the Jazan Region of Saudi Arabia and 38.12% prevalence reported by Jiang, X-L [27] but lower than the prevalence rate (83.3%) reported in the students of Ain Shams University in Cairo, Egypt by Ibrahim and Abouelezz [28].

The present study showed that genders had no significant impact on the severity and prevalence of insomnia. Many other studies also showed that gender had no impact on the severity and prevalence of insomnia [29–33]. In this study, the age factor had a high level of significance (p-value = 0.001) similar to the findings of a study conducted by Bhaskar, Hemavathy [34] in Bengaluru, India, and a study conducted by Cuadros, Fernández-Alonso [35]. Furthermore, the sector in which respondents worked (p value = 0.16) had no significant impact on the severity and prevalence of insomnia but other studies showed a positive link between the job sector and insomnia [36, 37]. Level of qualifications (p-value = 0.001) was positively linked with insomnia similar findings to the study conducted by Chen, Ying-Yeh [38] while many other studies showed a negative association between qualification level and prevalence of insomnia [36]. Furthermore, the healtcare workers demonstrated poor sleep quality, in particular during covid pandemic [39].

Sleep apnea a common type of sleep disorder was assessed in this study. Among all respondents, about 34.08% (n = 681) of respondents were at moderate to severe or severe levels of sleep apnea. The prevalence rate of sleep apnea was 34.08% higher than the prevalence rate (29.03) recorded in a study by Roche, Johanna [40].

The present study showed that gender had no significant impact on the severity and prevalence of sleep apnea however, many studies showed a statistically significant association between gender and sleep apnea [41]. The age factor was highly significant (p-value 0.001) in the severity and prevalence of sleep apnea as in other studies [42–44]. Furthermore, sector (p 0.206) had no significant impact on the severity and prevalence of sleep apnea but studies conducted on other professions such as drivers, or factory workers showed a positive link between profession and sleep apnea [45, 46]. An intriguing study presented data on the impact of continuous positive airway pressure (CPAP) treatment on reported HRQoL. Participants who reported more adherence to therapy, greater sleepiness, and greater improvement in daytime sleepiness as a result of CPAP therapy reported larger improvements in their overall quality of life. Gender comparisons reveal that males have superior perceived HRQoL at the time of their first visit and at the time of their CPAP follow-up, despite the fact that females have experienced a more significant improvement [19].

Gender had a significant impact on the occurrence of tension many other studies also showed similar results [47, 48]. A high significance (p-value 0.001) of the age factor for the occurrence of tension was also observed in this study and many other studies [36, 49, 50]. Furthermore, the sector (p-value 0.001) where respondents were working suggested a highly significant impact on the occurrence of tension [51–53]. The qualification level of respondents (p-value = 0.242) was not positively linked with the occurrence of tension. Poor sleep quality was associated significantly with higher stress levels. However, the relationship between academic performance has not been statistically significant [54].

The use of sleeping pills was increased with the increase in age. This study recorded the use of sleeping pills more in older age of the educational community. In chronic sleep disorders, patients use sleeping pills for falling asleep at night [55].

In the present study, it was recorded that sleep disorders either insomnia or sleep apnea was associated with many other health issues and diseases. Many other studies showed an association between sleep disorders and other health problems [56–59].

CBC test results showed a wide range differences between blood cells of a normal people and those who were suffering from a sleep disorders. High WBCs were recorded in sleep disorder patients in the present study similar finding to the results of other studies conducted earlier [60, 61]. RBCs were in the normal range however other studies showed a higher levels of RBCs in patients with sleep disorders [62]. Haemoglobin level (7.1–17 g/dl) was different from the lower reference range however from the upper reference rage was normal. HTC ranged between 22.2–47.5% which showed a lower level of haematocrit in sleepless patients similar to Liak and Fitzpatrick [63]. Increased red blood cell width distribution (RDW) (11.2–18.4) was recorded in the CBC test. Sleep duration and disturbed sleep are associated with elevated red blood cell distribution width [64]. Similarly increased platelet count (109–509 x10^3^/ μL) was recorded from CBC test as earlier studies mentioned that increased platelet count may be positively associated with sleep apnea [65]. Blood sugar level was also slightly increased ranging from 76–173. Diabetes is significantly associated with a sleep disorder [66]. Heartbeat level above the normal range was due to disturbed sleep [67].

In the present study sleep disorders were positively associated with headaches, migraines, depression, sugar or diabetes, obesity, myopia, and high blood pressure. While other studies also showed a positive association of sleep disorders with the above-mentioned diseases; headaches, Migraines [68–70], Depression [71, 72], diabetes [73, 74], obesity [75, 76], myopia [77] and high blood pressure [78]. Tension, depression, headache, and migraine were more associated with sleep disorders than all other health issues.

## Conclusions

This study concluded that individuals of the educational community were suffering from bad quality of sleep either short sleep duration or long sleep duration. Insomnia is more prevalent than sleep apnea among the educational community. Individuals of older age were using sleeping pills and also suffering from tension along with many other diseases like headaches, migraines, depression, diabetes, obesity, and myopia. Tension, depression, headache, and migraine were more associated with sleep disorders than all other health issues. This study recommended that as sleep disorders are neglected health problems and people are less aware to sleep health, Government should introduce sleep management programs and under these programs, government should create awareness related to the importance of sleep among general population.

## Supporting information

S1 File Questionnaire. (PDF)

S2 File Blood test reports. (PDF)

S3 File Raw data collected during survey. (XLSX)

S4 File Participant concent waived letter. (PDF)

## References

[1] How Much Sleep Do We Really Need? [Internet]. Sleep foundation. 2020 [cited 5 January, 2021]. Available from: https://www.sleepfoundation.org/how-sleep-works/how-much-sleep-do-we-really-need#:~:text=National%20Sleep%20Foundation%20guidelines1,to%208%20hours%20per%20night

[2] Sateia MJ. International classification of sleep disorders. Chest. 2014;146(5):1387–94. doi: 10.1378/chest.14-0970 25367475

[3] Stevens M. Normal sleep, sleep physiology, and sleep deprivation. Retrieved January 4, 2011, from the eMedicine Clinical Knowledge base. 2008.

[4] Pavlova MK, Latreille V. Sleep disorders. The American Journal of Medicine. 2019;132(3):292–9. 30292731 10.1016/j.amjmed.2018.09.021

[5] Vance DE, Heaton K, Eaves Y, Fazeli PL. Sleep and cognition on everyday functioning in older adults: implications for nursing practice and research. Journal of Neuroscience Nursing. 2011;43(5):261–71. doi: 10.1097/JNN.0b013e318227efb2 21926521

[6] Di Mauro P, Cocuzza S, Maniaci A, Ferlito S, Rasà D, Anzivino R, et al. The Effect of Adenotonsillectomy on Children’s Behavior and Cognitive Performance with Obstructive Sleep Apnea Syndrome: State of the Art. Children. 2021;8(10):921. doi: 10.3390/children8100921 34682186 PMC8535044

[7] Pasquali R, Colella P, Cirignotta F, Mondini S, Gerardi R, Buratti P, et al. Treatment of obese patients with obstructive sleep apnea syndrome (OSAS): effect of weight loss and interference of otorhinolaryngoiatric pathology. International journal of obesity. 1990;14(3):207–17. 2341227

[8] Krause AJ, Simon EB, Mander BA, Greer SM, Saletin JM, Goldstein-Piekarski AN, et al. The sleep-deprived human brain. Nature Reviews Neuroscience. 2017;18(7):404. doi: 10.1038/nrn.2017.55 28515433 PMC6143346

[9] Morgan D, Tsai SC. Sleep and the endocrine system. Critical care clinics 2015;31(3):403–18. 26118912 10.1016/j.ccc.2015.03.004

[10] Dyken ME, Afifi AK, Lin-Dyken DC. Sleep-related problems in neurologic diseases. Chest. 2012;141(2):528–44. doi: 10.1378/chest.11-0773 22315121

[11] Pace A, Iannella G, Rossetti V, Visconti IC, Gulotta G, Cavaliere C, et al. Diagnosis of obstructive sleep apnea in patients with allergic and non-allergic rhinitis. Medicina. 2020;56(9):454. doi: 10.3390/medicina56090454 32911862 PMC7559128

[12] Pollicina I, Maniaci A, Lechien JR, Iannella G, Vicini C, Cammaroto G, et al. Neurocognitive Performance Improvement after Obstructive Sleep Apnea Treatment: State of the Art. Behavioral Sciences. 2021;11(12):180. doi: 10.3390/bs11120180 34940115 PMC8698492

[13] Crowley K. Sleep and sleep disorders in older adults. Neuropsychology review. 2011;21(1):41–53. doi: 10.1007/s11065-010-9154-6 21225347

[14] Neikrug AB, Ancoli-Israel S. Sleep disorders in the older adult–a mini-review. Gerontology. 2010;56(2):181–9. doi: 10.1159/000236900 19738366 PMC2842167

[15] Saccomano SJ. Sleep disorders in older adults. Journal of gerontological nursing. 2014;40(3):38–45. doi: 10.3928/00989134-20131029-06 24219077

[16] Roepke SK, Ancoli-Israel SJ. Sleep disorders in the elderly. Indian J Med Res. 2010;131(2):302. 20308755

[17] Yaremchuk K. Sleep disorders in the elderly. Clinics in geriatric medicine. 2018;34(2):205–16. doi: 10.1016/j.cger.2018.01.008 29661333

[18] Foley Daniel, Ancoli-Israel S, Britz P, Walsh J. Sleep disturbances and chronic disease in older adults: results of the 2003 National Sleep Foundation Sleep in America Survey. Journal of psychosomatic research. 2004;56(5):497–502. doi: 10.1016/j.jpsychores.2004.02.010 15172205

[19] Chiu H-Y, Chen P-Y, Chuang L-P, Chen N-H, Tu Y-K, Hsieh Y-J, et al. Diagnostic accuracy of the Berlin questionnaire, STOP-BANG, STOP, and Epworth sleepiness scale in detecting obstructive sleep apnea: a bivariate meta-analysis. Sleep medicine reviews. 2017;36:57–70. 27919588 10.1016/j.smrv.2016.10.004

[20] Bastien CH, Vallières A, Morin CM. Validation of the Insomnia Severity Index as an outcome measure for insomnia research. Sleep medicine. 2001;2(4):297–307. doi: 10.1016/s1389-9457(00)00065-4 11438246

[21] Abrishami A, Khajehdehi A, Chung F. A systematic review of screening questionnaires for obstructive sleep apnea. Can J Anaesth. 2010;57(5):423–38. doi: 10.1007/s12630-010-9280-x 20143278

[22] Chen X, Wang S-B, Li X-L, Huang Z-H, Tan W-Y, Lin H-C, et al. Relationship between sleep duration and sociodemographic characteristics, mental health and chronic diseases in individuals aged from 18 to 85 years old in Guangdong province in China: a population-based cross-sectional study. BMC psychiatry. 2020;20(1):1–10. 32938430 10.1186/s12888-020-02866-9 PMC7493355

[23] Liang Y, Qu L-B, Liu H. Non-Linear associations between sleep duration and the risks of mild cognitive impairment/dementia and cognitive decline: a dose–response meta-analysis of observational studies. Aging clinical experimental research. 2019;31(3):309–20. doi: 10.1007/s40520-018-1005-y 30039452

[24] Gonzaga C, Bertolami A, Bertolami M, Amodeo C, Calhoun D. Obstructive sleep apnea, hypertension and cardiovascular diseases. Journal of human hypertension. 2015;29(12):705–12. doi: 10.1038/jhh.2015.15 25761667

[25] Fan L, Xu W, Cai Y, Hu Y, Wu C. Sleep duration and the risk of dementia: A systematic review and meta-analysis of prospective cohort studies. Journal of the American Medical Directors Association. 2019;20(12):1480–7. e5. doi: 10.1016/j.jamda.2019.06.009 31604673

[26] Albasheer OB, Al Bahhawi T, Ryani MA, Arishi AM, Hakami OM, Maashi SM, et al. Prevalence of insomnia and relationship with depression, anxiety and stress among Jazan University students: A cross-sectional study. Cogent Psychology. 2020;7(1):1789424.

[27] Jiang X-L, Zheng X-Y, Yang J, Ye C-p, Chen Y-Y, et al. A systematic review of studies on the prevalence of insomnia in university students. Public health. 2015;129(12):1579–84. doi: 10.1016/j.puhe.2015.07.030 26298588

[28] Ibrahim JM, Abouelezz NF. Relationship between insomnia and computer use among students at Ain Shams University, Cairo, Egypt, Egypt. J. Country: Egypt. 2011;29(2):31–9.

[29] Foley J D, Monjan AA, Brown SL, Simonsick EM, Wallace RB, et al. Sleep complaints among elderly persons: an epidemiologic study of three communities. Sleep. 1995;18(6):425–32. doi: 10.1093/sleep/18.6.425 7481413

[30] Foley J D, Monjan A, Simonsick EM, Wallace RB, Blazer DG. Incidence and remission of insomnia among elderly adults: an epidemiologic study of 6,800 persons over three years. Sleep: Journal of Sleep Research Sleep Medicine. 1999. 10394609

[31] Sivertsen B, Krokstad S, Øverland S, Mykletun A. The epidemiology of insomnia: Associations with physical and mental health.: The HUNT-2 study. Journal of psychosomatic research. 2009;67(2):109–16. doi: 10.1016/j.jpsychores.2009.05.001 19616137

[32] Gu D, Sautter J, Pipkin R, Zeng Y. Sociodemographic and health correlates of sleep quality and duration among very old Chinese. Sleep. 2010;33(5):601–10. doi: 10.1093/sleep/33.5.601 20469802 PMC2864875

[33] Dragioti E, Levin L-Å, Bernfort L, Larsson B, Gerdle B. Insomnia severity and its relationship with demographics, pain features, anxiety, and depression in older adults with and without pain: cross-sectional population-based results from the PainS65+ cohort. Annals of general psychiatry. 2017;16(1):1–10. 28250802 10.1186/s12991-017-0137-3 PMC5324239

[34] Bhaskar S, Hemavathy D, Prasad S. Prevalence of chronic insomnia in adult patients and its correlation with medical comorbidities. Journal of family medicine primary care. 2016;5(4):780. doi: 10.4103/2249-4863.201153 28348990 PMC5353813

[35] Cuadros JL, Fernández-Alonso AM, Cuadros-Celorrio ÁM, Fernández-Luzón N, Guadix-Peinado MJ, del Cid-Martín N, et al. Perceived stress, insomnia and related factors in women around the menopause. Maturitas. 2012;72(4):367–72. doi: 10.1016/j.maturitas.2012.05.012 22721806

[36] Kim, Min B, Jung J, Paek D, Cho S-i. The association of relational and organizational job stress factors with sleep disorder: analysis of the 3rd Korean working conditions survey (2011). Annals of Occupational Environmental Medicine. 2016;28(1):1–11. 10.1186/s40557-016-0131-2 PMC5020473 27625788

[37] Nauman S, Malik SZ, Jalil F. How workplace bullying jeopardizes employees’ life satisfaction: The roles of job anxiety and insomnia. Frontiers in psychology. 2019;10:2292. doi: 10.3389/fpsyg.2019.02292 31708827 PMC6821672

[38] Chen Ying-Yeh, Kawachi I, Subramanian S, Acevedo-Garcia D, Lee Y-J. Can social factors explain sex differences in insomnia? Findings from a national survey in Taiwan. ournal of Epidemiology Community Health. 2005;59(6):488–94. doi: 10.1136/jech.2004.020511 15911645 PMC1757060

[39] Maniaci A, Ferlito S, Bubbico L, Ledda C, Rapisarda V, Iannella G, et al. Comfort rules for face masks among healthcare workers during COVID-19 spread. Ann Ig. 2021:615–27. doi: 10.7416/ai.2021.2439 33797548

[40] Roche Johanna, Rae D, Redman K, Knutson K, von Schantz M, et al. Impact of obstructive sleep apnea on cardiometabolic health in an aging population in rural South Africa: Building the case for the treatment of sleep disorders in deprived settings. medRxiv. 2020. 10.5664/jcsm.9214 PMC8314613 33687325

[41] Gabbay IE, Lavie P. Age-and gender-related characteristics of obstructive sleep apnea. Sleep Breathing. 2012;16(2):453–60. doi: 10.1007/s11325-011-0523-z 21499842

[42] Hirotsu C, Albuquerque RG, Nogueira H, Hachul H, Bittencourt L, Tufik S, et al. The relationship between sleep apnea, metabolic dysfunction and inflammation: the gender influence. Brain, behavior, immunity. 2017;59:211–8. doi: 10.1016/j.bbi.2016.09.005 27621224

[43] Nigro CA, Dibur E, Borsini E, Malnis S, Ernst G, Bledel I, et al. The influence of gender on symptoms associated with obstructive sleep apnea. Sleep Breathing. 2018;22(3):683–93. doi: 10.1007/s11325-017-1612-4 29392572

[44] Seguin L, Tamisier R, Deletombe B, Lopez M, Pepin J-L, Payen J-F. Preoperative screening for obstructive sleep apnea using alternative scoring models of the sleep tiredness observed pressure-body mass index age neck circumference gender questionnaire: an external validation. Anesthesia Analgesia. 2020;131(4):1025–31. doi: 10.1213/ANE.0000000000004909 32925319

[45] Eckert DJ, Sweetman A. Impaired central control of sleep depth propensity as a common mechanism for excessive overnight wake time: Implications for sleep apnea, insomnia and beyond. Journal of Clinical Sleep Medicine. 2020;16(3):341–3. doi: 10.5664/jcsm.8268 32003739 PMC7075088

[46] Mulgrew A, Ryan C, Fleetham J, Cheema R, Fox N, Koehoorn M, et al. The impact of obstructive sleep apnea and daytime sleepiness on work limitation. Sleep medicine. 2007;9(1):42–53. doi: 10.1016/j.sleep.2007.01.009 17825611

[47] Rasmussen BK. Migraine and tension-type headache in a general population: precipitating factors, female hormones, sleep pattern and relation to lifestyle. Pain. 1993;53(1):65–72. 8316392 10.1016/0304-3959(93)90057-V

[48] Li X, Gao X, Liu J. Cross-sectional survey on the relationship between occupational stress, hormone levels, and the sleep quality of oilfield workers in Xinjiang, China. International journal of environmental research public health. 2019;16(18):3316. 10.3390/ijerph16183316 PMC6765891 31505823

[49] Fu W, Wang C, Zou L, Guo Y, Lu Z, Yan S, et al. Psychological health, sleep quality, and coping styles to stress facing the COVID-19 in Wuhan, China. Translational psychiatry. 2020;10(1):1–9. 32647160 10.1038/s41398-020-00913-3 PMC7347261

[50] Elliott JE, Opel RA, Pleshakov D, Rachakonda T, Chau AQ, Weymann KB, et al. Posttraumatic stress disorder increases the odds of REM sleep behavior disorder and other parasomnias in Veterans with and without comorbid traumatic brain injury. Sleep. 2020;43(3):zsz237. doi: 10.1093/sleep/zsz237 31587047 PMC7315766

[51] Kalimo R, Tenkanen L, Härmä M, Poppius E, Heinsalmi P. Job stress and sleep disorders: findings from the Helsinki Heart Study. Stress Medicine. 2000;16(2):65–75.

[52] Åkerstedt T, Knutsson A, Westerholm P, Theorell T, Alfredsson L, Kecklund G. Sleep disturbances, work stress and work hours: a cross-sectional study. Journal of psychosomatic research. 2002;53(3):741–8. doi: 10.1016/s0022-3999(02)00333-1 12217447

[53] Ursin R, Baste V, Moen BE. Sleep duration and sleep-related problems in different occupations in the Hordaland Health Study. candinavian journal of work, environment health. 2009:193–202. doi: 10.5271/sjweh.1325 19436924

[54] Alotaibi AD, Alosaimi FM, Alajlan AA, Abdulrahman KAB. The relationship between sleep quality, stress, and academic performance among medical students. Journal of family community medicine. 2020;27(1):23. 32030075 10.4103/jfcm.JFCM_132_19 PMC6984036

[55] McCall WV. Sleep in the elderly: burden, diagnosis, and treatment. Primary care companion to the Journal of clinical psychiatry. 2004;6(1):9. 10.4088/pcc.v06n0104 PMC427621 15486596

[56] Somers VK, White DP, Amin R, Abraham WT, Costa F, Culebras A, et al. Sleep apnea and cardiovascular disease: An American heart association/American college of cardiology foundation scientific statement from the American heart association council for high blood pressure research professional education committee, council on clinical cardiology, stroke council, and council on cardiovascular nursing in collaboration with the national heart, lung, and blood institute national center on sleep disorders research (national institutes of health). Journal of the American College of Cardiology. 2008;52(8):686–717. 18702977 10.1016/j.jacc.2008.05.002

[57] Fang H, Tu S, Sheng J, Shao A. Depression in sleep disturbance: a review on a bidirectional relationship, mechanisms and treatment. Journal of cellular molecular medicine. 2019;23(4):2324–32. doi: 10.1111/jcmm.14170 30734486 PMC6433686

[58] Medic G, Wille M, Hemels ME. Short-and long-term health consequences of sleep disruption. Nature science of sleep. 2017;9:151. doi: 10.2147/NSS.S134864 28579842 PMC5449130

[59] Brzecka A, Leszek J, Ashraf GM, Ejma M, Ávila-Rodriguez MF, Yarla NS, et al. Sleep disorders associated with Alzheimer’s disease: a perspective. Frontiers in neuroscience. 2018;12:330. doi: 10.3389/fnins.2018.00330 29904334 PMC5990625

[60] Boudjeltia KZ, Faraut B, Stenuit P, Esposito MJ, Dyzma M, Brohée D, et al. Sleep restriction increases white blood cells, mainly neutrophil count, in young healthy men: a pilot study. J Vascular health risk management. 2008;4(6):1467. doi: 10.2147/vhrm.s3934 19337560 PMC2663438

[61] Kerkhofs M, Boudjeltia KZ, Stenuit P, Brohée D, Cauchie P, Vanhaeverbeek M. Sleep restriction increases blood neutrophils, total cholesterol and low density lipoprotein cholesterol in postmenopausal women: a preliminary study. Maturitas. 2007;56(2):212–5. doi: 10.1016/j.maturitas.2006.07.007 16950577

[62] Khalyfa A, Sanz-Rubio D. The Mystery of Red Blood Cells Extracellular Vesicles in Sleep Apnea with Metabolic Dysfunction. International Journal of Molecular Sciences. 2021;22(9):4301. doi: 10.3390/ijms22094301 33919065 PMC8122484

[63] Liak C, Fitzpatrick M. Coagulability in obstructive sleep apnea. Canadian respiratory journal. 2011;18(6):338–48. doi: 10.1155/2011/924629 22187690 PMC3267624

[64] Loprinzi PD. Sleep duration and sleep disorder with red blood cell distribution width. American journal of health behavior. 2015;39(4):471–4. doi: 10.5993/AJHB.39.4.3 26018095

[65] Sökücü SN, Özdemir C, Dalar L, Karasulu L, Aydın Ş, Altın S. Is mean platelet volume really a severity marker for obstructive sleep apnea syndrome without comorbidities? Pulmonary medicine. 2014;2014. doi: 10.1155/2014/754839 25309752 PMC4182073

[66] Khorasani ZM, Ravan VR, Hejazi S. Evaluation of the prevalence of sleep disorder among patients with type 2 diabetes mellitus referring to Ghaem hospital from 2016 to 2017. Current Diabetes Reviews. 2021;17(2):214–21. doi: 10.2174/1573399816666200527140340 32459608

[67] Perogamvros L, Park H-D, Bayer L, Perrault AA, Blanke O, Schwartz S. Increased heartbeat-evoked potential during REM sleep in nightmare disorder. NeuroImage: Clinical. 2019;22:101701. doi: 10.1016/j.nicl.2019.101701 30739843 PMC6370851

[68] Kim Han K-T, Jang S-Y, Yoo K-B, Kim SJ. The association between migraine and types of sleep disorder. International journal of environmental research public health. 2018;15(12):2648. doi: 10.3390/ijerph15122648 30486273 PMC6313424

[69] Bruni O, Fabrizi P, Ottaviano S, Cortesi F, Giannotti F, Guidetti V. Prevalence of sleep disorders in childhood and adolescence with headache: a case-control study. Cephalalgia. 1997;17(4):492–8. doi: 10.1046/j.1468-2982.1997.1704492.x 9209768

[70] Sancisi E, Cevoli S, Vignatelli L, Nicodemo M, Pierangeli G, Zanigni S, et al. Increased prevalence of sleep disorders in chronic headache: a case–control study. Headache: The Journal of Head Face Pain. 2010;50(9):1464–72. doi: 10.1111/j.1526-4610.2010.01711.x 20572880

[71] Wang Xiaofen, Cheng S, Xu H. Systematic review and meta-analysis of the relationship between sleep disorders and suicidal behaviour in patients with depression. BMC Psychiatry. 2019;19(1):1–13. doi: 10.1186/s12888-019-2302-5 31623600 PMC6798511

[72] Roberts RE, Duong HT. The prospective association between sleep deprivation and depression among adolescents. Sleep. 2014;37(2):239–44. doi: 10.5665/sleep.3388 24497652 PMC3900610

[73] Engeda J, Mezuk B, Ratliff S, Ning Y. Association between duration and quality of sleep and the risk of pre‐diabetes: evidence from NHANES. Diabetic Medicine. 2013;30(6):676–80. doi: 10.1111/dme.12165 23425048 PMC3660430

[74] Khalil Marina, Power N, Graham E, Deschênes SS, Schmitz N. The association between sleep and diabetes outcomes–A systematic review. Diabetes research clinical practice. 2020;161:108035. doi: 10.1016/j.diabres.2020.108035 32006640

[75] Naufel MF, Frange C, Andersen ML, Girão MJBC, Tufik S, Beraldi Ribeiro E, et al. Association between obesity and sleep disorders in postmenopausal women. Menopause. 2018;25(2):139–44. doi: 10.1097/GME.0000000000000962 28926516

[76] Litsfeldt S, Ward TM, Hagell P, Garmy P. Association between sleep duration, obesity, and school failure among adolescents. The Journal of School Nursing. 2020;36(6):458–63. 31973678 10.1177/1059840520901335 PMC7675773

[77] Zhou Z, Morgan IG, Chen Q, Jin L, He M, Congdon N. Disordered sleep and myopia risk among Chinese children. PloS one. 2015;10(3):e0121796. doi: 10.1371/journal.pone.0121796 25811755 PMC4374782

[78] Jiang, Hu C, Li F, Hua X, Zhang X. Association between sleep duration and high blood pressure in adolescents: a systematic review and meta-analysis. Annals of human biology. 2018;45(6–8):457–62. doi: 10.1080/03014460.2018.1535661 30387692

