# Supplementary material for: Epidemiological studies of sleep disorder in educational community of Pakistani population, its major risk factors and associated diseases
Source: PLoS One. 2022 Apr 21;17(4):e0266739. doi: 10.1371/journal.pone.0266739 (PMC9022811; doi:10.1371/journal.pone.0266739)
Supplement: S1 File — (PDF) [file pone.0266739.s001.pdf]

Epidemiologic studies of sleep

I am a M.Phil. research student. I am conducting a survey about "Sleep and its Quality among Educational Community" you have been conducted to provide information. Your responses shall be helpful to achieve objectives of this study. I assure you that your participant shall be kept confidence and information provided shall be used for study purpose only. I am thankful to you for spending some time to fill this questionnaire. Thank you for your participation.

* Required

1. Name *

2. Contact information *

3. Gender *

Mark only one oval.

- Male
- Female

4. Age *

Mark only one oval.

- 25 or less than 25 Years
- 26-35 Years
- 36-45 Years
- More than 45 Years

5. Your Qualifications *

Mark only one oval.

- [ ] Graduation
- [ ] Post Graduation

6. Where You are working? *

Mark only one oval.

- [ ] School
- [ ] College
- [ ] University

7. You are employee in which sector? *

Mark only one oval.

- [ ] Government
- [ ] Private

8. At sleeping time, you sleep *

Mark only one oval.

- [ ] Immediately
- [ ] Took short time
- [ ] Took long time

9. Usually you sleep *

Mark only one oval.

- Less than 7 hours
- 7-8 hours
- More than 8 hours

10. During Sleeping, you sleep *

Mark only one oval.

- Continuously
- Awake one time
- Awake 2 or more times

11. Do you snore in sleep *

Mark only one oval.

- Yes
- No
- Some times

12. What you feel upon waking in morning? *

Mark only one oval.

- Refreshed
- Un refreshed
- Tired

13. During the day you feel.... *

Mark only one oval.

- Sleepy/tired/irritable
- Moderately active
- Fresh and alert

14. Often get told by others that you look tired. *

Mark only one oval.

- Agree
- Disagree
- Sometimes

15. Mood during daytime *

Mark only one oval.

- Good
- Average
- Bad

16. Do you require tea to keep yourself refresh and active? *

Mark only one oval.

- Agree
- Disagree
- Sometimes

17. Usually blood pressure remains *

Mark only one oval.

- Normal
- Below normal
- Above normal

18. Suffering from tension *

Mark only one oval.

- Yes
- No
- Not now but suffered in past

19. Do you take sleeping pills at night? *

Mark only one oval.

- Yes
- No
- Sometimes

20. Do you feel irritating during day? *

Mark only one oval.

- Yes
- No
- Sometimes

21. Are you suffering from any one from following diseases? *

Mark only one oval.

- Headache or Migraine
- Obesity
- Depression
- Sugar/Diabetes
- Heart problem
- Liver problem
- Stomach Problem
- Myopia
- Asthma
- Others
- Not Suffering

This content is neither created nor endorsed by Google.

Google Forms
