# Supplementary material for: Epidemiological studies of sleep disorder in educational community of Pakistani population, its major risk factors and associated diseases
Source: PLoS One. 2022 Apr 21;17(4):e0266739. doi: 10.1371/journal.pone.0266739 (PMC9022811; doi:10.1371/journal.pone.0266739)
Supplement: S2 File — (PDF) [file pone.0266739.s002.pdf]

Khair ul Bashar Medical Center
Test Report

No.: 4
Name: ALLAH DITTA
Sex: Male Age:
Type: Whole blood
Reference: General
Medical No.: Bed No.:
Dept.: Applicant:
Inspector: Verifier:
Test Time: 02/01/2021 11:13:07

Item	Result	Reference	Note
WBC	10.03 10^3/uL	4.00-10.00	H
LYM#	2.35 10^3/uL	0.60-4.10	
MID#	0.57 10^3/uL	0.10-0.90	
GRA#	7.11 10^3/uL	2.00-7.80	
LYM%	23.5 %	20.0-50.0	
MID%	5.7 %	3.0-10.0	
GRA%	70.8 %	40.0-70.0	H
RBC	3.83 10^12/L	3.80-5.80	
HGB	7.1 g/dL	11.0-16.5	L-
MCHC	32.0 g/dL	32.0-36.0	
MCH	18.6 pg	26.5-33.5	L-
MCV	58.1 fL	80.0-99.0	L-
RDW-CV	12.9 %	10.0-15.0	
RDW-SD	35.0 fL	35.0-56.0	
HCT	22.2 %	35.0-50.0	L-
PLT	509 10^3/uL	100-300	H+
MPV	8.3 fL	7.0-11.0	
PDW	13.7 %	10.0-18.0	
PCT	0.424 %	0.100-0.500	
P-LCR	16.4 %	13.0-43.0	

Print Date: 02/01/2021

Scanned with CamScanner

H # F-833/3 Opp: Gate # 2
Holy Family Hospital
Rawalpindi

The Molecular Concerns
Reliable DNA Translationists

051-87[illegible]
051-8742433
themolecularconcern[illegible]
PHC. Reg. No 50653

Patient Name:	*ALLHA DITTA*	*Date:*	*01/01/2021*
Lab #.	*001102*	*Ag/Sex*	*Y/M*
Refer By.	**SELF**		

Hematology Department

(CBC TEST)

Test	Result	Unit	Normal Value
**Haemoglobin**	*7.1*	**G/dl**	**Male 14-18 Female 12-16**
**Total Leucocytes Count.**	***10,300***	**Cmm**	**4000 – 11000**
**Platelet Count**	*5,00,000*	**Cmm**	**150, 000 – 450,000**
**Neutrophil**	*70*	**%**	**55 – 70**
**Lymphocytes**	*23*	**%**	**25 – 40**
**Monocytes**	*05*	**%**	**02 – 07**
**Eosinophils**	*02*	**%**	**01 – 05**
**RBC**	*3.8*	**x10.e6/ul**	**4----6**
**HCT**	*22.2*	**%**	**36----54**
**MCV**	*58.1*	**Fl**	**76----96**
**MCH**	*18.6*	**Pg**	**27----33**
**MCHC**	*32.0*	**g/dl**	**33----35**

Dr. Mehreen Fatima
M.B.B.S, M.Phil
Consultant Histopathologist

M. Usman
PhD Scholar Microbiology

M. Iqbal
B.Sc MLT
M.Sc Microbiology

M. Umer
M Phil Molecular Pathology and Cytogenetics

Muhammad Uzair Mansoor
PhD Scholar Biotechnology
M.S Molecular Virology

Dr. Mehran Kausar
Ph.D (Biochemistry & Molecular Biology)
Consultant Molecular Biologist

Not for any legal official purpose

Scanned with CamScanner

H # F-833/3 Opp: Gate # 2
Holy Family Hospital
Rawalpindi

The Molecular Concerns

Reliable DNA Translationists

051-[illegible]
051-8[illegible]
themolec[illegible]
PHC. Reg.

Patient Name:	ALLHA DITTA	Date:	01/01/2021
Lab #.	001102	Age / Sex	Y/M
Refer By.	SELF		

Biochemistry Department

Test	Result	Unit	Normal Value
B.SUGAR 'R'	111	Mg/dl	80-----140
S.Uric Acid	5.5	Mg/dl	F: 2.3 – 5.7<br>M: 3.5 – 7.0

Dr. Mehreen Fatima
M.B.B.S, M.Phil
Consultant Histopathologist

M. Usman
PhD Scholar Microbiology

M. Iqbal
B.Sc MLT
M.Sc Microbiology

M. Umer
M Phil Molecular Pathology and Cytogenetics

Muhammad Uzair Mansoor
PhD Scholar Biotechnology
M.S Molecular Virology

Dr. Mehran Kausar
Ph.D (Biochemistry & Molecular Biology)
Consultant Molecular Biologist

Not for any legal official purpose

Scanned with CamScanner

[illegible] Laboratory
[illegible] Sargodha Road, [illegible] 041-8869861 Ext.116 , 172

LAB REPORT

ACCN. NO. **20-303-0157**

[illegible]me : ALLAH MAFI W/O M SAJID AMIN
[illegible]ex : Female Age : 33 Y OPD Slip/MR # : 2010290559 0120087110
[illegible]NIC. 0110114467684 Phone # : 3454912171
[illegible]ress: Po Mamokangan Chak No 514 G B Chak Tehsil Ta Requesting Physician : N/A
[illegible]e # : 1526905 Bed # : Cash Receipt # : 1945862 Requested By : OPD

HAEMATOLOGY

COLL: 29/10/2020 - 14:05 RECD: 29/10/2020 - 14:05
VERIFY: 29/10/2020 - 14:20 VERIFY BY: DR. SADIA IJAZ

[illegible]C *Complete Blood Count*

Test	Result	Unit	Reference Values
WBC White Blood Cells	**14.6H**	x 10 ^ 9 / L	4.0 - 11.0
RBC Red Blood Cells	5.27	x 10 ^ 12 / L	3.8 - 5.8
HGB Haemoglobin	14.7	g / dL	11.5 - 16.5
HCT Haematocrit	43	%	34.0 - 47.0
MCV Mean Cell Volume	82	fL	75.0 - 95.0
MCH Mean Cell Haemoglobin	27.9	pg	24.0 - 32.0
MCHC Mean Cell Haemoglobin Concentration	34.1	g / dL	31.0 - 35.0
PLT Platelet Count	214	x 10 ^ 9 / L	150.0 - 450.0
MPV Mean Platelet Volume	10.6	fL	7.8 - 11.0
RDW Red Cell Distribution Width	**15.2H**	%	11.0 - 14.0
NEUT Neutrophil Count	**9.94H**	x 10 ^ 9 / L	1.8 - 7.5
LYMPH Lymphocyte Count	3.65	x 10 ^ 9 / L	1.5 - 4.0
MONO Monocyte Count	**0.96H**	x 10 ^ 9 / L	0.2 - 0.8
EOS Eosinophil Count	0.05	x 10 ^ 9 / L	0.0 - 0.6
BASO Basophil Count	0.03	x 10 ^ 9 / L	0.0 - 0.1

Dr Sadia Hameed
MBBS DCP MPhil
(Histopath)

Dr Arif Hussain
MBBS MCPS MPhil
(Haematology)

Dr Shazia Aslam
MBBS MPhil
(Histopath)

Dr Sadia Ijaz
MBBS MPhil
[illegible]

Scanned with CamScanner

H # F-833/3 Opp: Gate # 2
Holy Family Hospital
Rawalpindi

The Molecular Concerns

Reliable DNA Translationists

051-87[illegible]
051-8742433
themolecularconcernslab[illegible]
PHC. Reg. No 50653

Patient Name:	ALI UMAR	Date:	01/01/2021
Lab #.	001106	Ag/Sex	Y/M
Refer By.	SELF		

Hematology Department

(CBC TEST)

Test	Result	Unit	Normal Value
**Haemoglobin**	16.2	G/dl	Male 14-18 Female 12-16
**Total Leucocytes Count.**	14,600	Cmm	4000 – 11000
**Platelet Count**	2,80,000	Cmm	150, 000 – 450,000
**Neutrophil**	58	%	55 – 70
**Lymphocytes**	37	%	25 – 40
**Monocytes**	04	%	02 – 07
**Eosinophils**	01	%	01 – 05
**RBC**	5.4	x10.e6/ul	4-----6
**HCT**	47.5	%	36----54
**MCV**	87.1	Fl	76----96
**MCH**	31.2	Pg	27----33
**MCHC**	35.8	g/dl	33----35

Dr. Mehreen Fatima
M.B.B.S, M.Phil
Consultant Histopathologist

M. Usman
PhD Scholar Microbiology

M. Iqbal
B.Sc MLT
M.Sc Microbiology

M. Umer
M Phil Molecular Pathology
and Cytogenetics

Muhammad Uzair Mansoor
PhD Scholar Biotechnology
M.S Molecular Virology

Dr. Mehran Kausar
Ph.D (Biochemistry & Molecular Biology)
Consultant Molecular Biologist

Not for any legal official purpose

Scanned with CamScanner

H # F-833/3 Opp: Gate # 2
Holy Family Hospital
Rawalpindi

The Molecular Concerns

Reliable DNA Translationists

051-8742432
051-8742433
themolecularconcernslab@gm[illegible]
PHC. Reg. No 50653

**Patient Name:**	*ALI UMAR*	**Date:**	*01/01/2021*
**Lab #.**	*001106*	**Age / Sex**	*Y/M*
**Refer By.**	*SELF*		

Biochemistry Department

Test	Result	Unit	Normal Value
B.SUGAR 'R'	129	*Mg/dl*	80-----140
S.Uric Acid	4.8	Mg/dl	F: 2.3 – 5.7<br>M: 3.5 – 7.0

Dr. Mehreen Fatima
M.B.B.S, M.Phil
Consultant Histopathologist

M. Usman
PhD Scholar Microbiology

M. Iqbal
B.Sc MLT
M.Sc Microbiology

M. Umer
M Phil Molecular Pathology
and Cytogenetics

Muhammad Uzair Mansoor
PhD Scholar Biotechnology
M.S Molecular Virology

Dr. Mehran Kausar
Ph.D (Biochemistry & Molecular Biology)
Consultant Molecular Biologist

Not for any legal official purpose

Scanned with CamScanner

H # F-833/3 Opp: Gate # 2
Holy Family Hospital
Rawalpindi

The Molecular Concerns

Reliable DNA Translationists

051-8742432
051-8742433
themolecularconcernslab@gmail.com
PHC. Reg. No 50653

Patient Name:	*MR.KAMRAN*	*Date:*	*01/01/2021*
Lab #.	*001101*	*Ag/Sex*	*Y/M*
Refer By.	**SELF**		

Hematology Department

(CBC TEST)

Test	Result	Unit	Normal Value
**Haemoglobin**	*13.4*	**G/dl**	**Male 14-18 Female 12-16**
**Total Leucocytes Count.**	***8,700***	**Cmm**	**4000 – 11000**
**Platelet Count**	*2,20,000*	**Cmm**	**150, 000 – 450,000**
**Neutrophil**	*55*	**%**	**55 – 70**
**Lymphocytes**	*35*	**%**	**25 – 40**
**Monocytes**	*08*	**%**	**02 – 07**
**Eosinophils**	*02*	**%**	**01 – 05**
**RBC**	*5.1*	**x10.e6/ul**	**4-----6**
**HCT**	*46.4*	**%**	**36----54**
**MCV**	*84.5*	**Fl**	**76----96**
**MCH**	*27.0*	**Pg**	**27----33**
**MCHC**	*22.5*	**g/dl**	**33----35**
**E.S.R.**	*14*	**mm. in 1st hour**	**Male: 4 – 10 Female. 4– 20**

Dr. Mehreen Fatima
M.B.B.S, M.Phil
Consultant Histopathologist

M. Usman
PhD Scholar Microbiology

M. Iqbal
B.Sc MLT
M.Sc Microbiology

M. Umer
M Phil Molecular Pathology
and Cytogenetics

Muhammad Uzair Mansoor
PhD Scholar Biotechnology
M.S Molecular Virology

Dr. Mehran Kausar
Ph.D (Biochemistry & Molecular Biology)
Consultant Molecular Biologist

Not for any legal official purpose

Scanned with CamScanner

H # F-833/3 Opp: Gate # 2
Holy Family Hospital
Rawalpindi

051-8742432
051-8742433
themolecularconcern
PHC. Reg. No 50653

The Molecular Concerns

Reliable DNA Translationists

Patient Name:	MR.KAMRAN	Date:	01/01/2021
Lab #.	001101	Age / Sex	Y/M
Refer By.	SELF		

Biochemistry Department

Test	Result	Unit	Normal Value
B.SUGAR 'R'	173	Mg/dl	80-----140
S.Uric Acid	5.9	Mg/dl	F: 2.3 – 5.7<br>M: 3.5 – 7.0

Dr. Mehreen Fatima
M.B.B.S, M.Phil
Consultant Histopathologist

M. Usman
PhD Scholar Microbiology

M. Iqbal
B.Sc MLT
M.Sc Microbiology

M. Umer
M Phil Molecular Pathology
and Cytogenetics

Muhammad Uzair Mansoor
PhD Scholar Biotechnology
M.S Molecular Virology

Dr. Mehran Kausar
Ph.D (Biochemistry & Molecular Biology)
Consultant Molecular Biologist

Not for any legal official purpose

Scanned with CamScanner

H # F-833/3 Opp: Gate # 2
Holy Family Hospital
Rawalpindi

051-8742432
051-8742433
themolecularconcernsla
PHC. Reg. No 50653

The Molecular Concerns

Reliable DNA Translationists

Patient Name:	MR.AMIN	Date:	01/01/2021
Lab #.	001104	Ag/Sex	Y/M
Refer By.	SELF		

Hematology Department

(CBC TEST)

Test	Result	Unit	Normal Value
**Haemoglobin**	*10.1*	**G/dl**	**Male 14-18 Female 12-16**
**Total Leucocytes Count.**	***7,500***	**Cmm**	**4000 -- 11000**
**Platelet Count**	*4,10,000*	**Cmm**	**150, 000 – 450,000**
**Neutrophil**	*70*	**%**	**55 – 70**
**Lymphocytes**	*23*	**%**	**25 – 40**
**Monocytes**	*06*	**%**	**02 – 07**
**Eosinophils**	*01*	**%**	**01 – 05**
**RBC**	*5.1*	**x10.e6/ul**	**4-----6**
**HCT**	*30.1*	**%**	**36----54**
**MCV**	*60.1*	**Fl**	**76----96**
**MCH**	*20.2*	**Pg**	**27----33**
**MCHC**	*33.5*	**g/dl**	**33----35**

Dr. Mehreen Fatima
M.B.B.S, M.Phil
Consultant Histopathologist

M. Usman
PhD Scholar Microbiology

M. Iqbal
B.Sc MLT
M.Sc Microbiology

M. Umer
M Phil Molecular Pathology and Cytogenetics

Muhammad Uzair Mansoor
PhD Scholar Biotechnology
M.S Molecular Virology

Dr. Mehran Kausar
Ph.D (Biochemistry & Molecular Biology)
Consultant Molecular Biologist

Not for any legal official purpose

Scanned with CamScanner

H # F-833/3 Opp: Gate # 2
Holy Family Hospital
Rawalpindi

051-8742432
051-8742433
themolecularconcernslab
PHC. Reg. No 50653

The Molecular Concerns

Reliable DNA Translationists

Patient Name:	MR:AMIN	Date:	01/01/2021
Lab #.	001104	Age / Sex	Y/M
Refer By.	SELF		

Biochemistry Department

Test	Result	Unit	Normal Value
B.SUGAR 'R'	109	Mg/dl	80-----140
S.Uric Acid	5.2	Mg/dl	F: 2.3 – 5.7<br>M: 3.5 – 7.0

Dr. Mehreen Fatima
M.B.B.S, M.Phil
Consultant Histopathologist

M. Usman
PhD Scholar Microbiology

M. Iqbal
B.Sc MLT
M.Sc Microbiology

M. Umer
M Phil Molecular Pathology and Cytogenetics

Muhammad Uzair Mansoor
PhD Scholar Biotechnology
M.S Molecular Virology

Dr. Mehran Kausar
Ph.D (Biochemistry & Molecular Biology)
Consultant Molecular Biologist

Not for any legal official purpose

Scanned with CamScanner

MADINAH TEACHING HOSPITAL (M.T.H)

Department of Medical Laboratory
Sargodha Road, Faisalabad. Tel: 041-8869861 Ext.116, 172

PBTA License No : 017

LAB REPORT

Name : TANZEELA AHMAD W/O AHMAD JAHANZAIB

Sex : Female Age :24 Y OPD Slip/MR # :2007100306 0120017171

CNIC 3310321697860 Phone # : 3007144105

ACCN. NO. **20-197-0087**

Address Fsd Requesting Physician : N/A

File # Bed # : Cash Receipt # : 1890777 Requested By : OPD

Test	Result	Unit	Reference Values

CLINICAL CHEMISTRY

Diabetic Monitoring

COLL: 15/07/2020 - 11:47 RECD: 15/07/2020 11:47
VERIFY: 15/07/2020 - 12:36 VERIFY BY:DR. SADIA IJAZ

Test	Result	Unit	Reference Values
RBS Blood Sugar Random	93.00	mg / dL	80.0 - 130.0

Dr. Asif Hussain Dr.Shazia Aslam Dr.Sadia Ijaz

Scanned with CamScanner

MADINAH TEACHING HOSPITAL (M T H)

Department of Medical Laboratory

PSTA License No : 017 Sargodha Road, Faisalabad. Tel: 041-8869861 Ext.116, 172

LAB REPORT

Name : TANZEELA AHMAD W/O AHMAD JAHANZAIB.
Sex : Female Age :24 Y OPD Slip/MR # :2010140412 0120017171
CNIC. 3310321697860 Phone # :3007144105

ACCN. NO.
20-288-0130

Address: Chak No 05 Kamal Pur Dist Fsd Requesting Physician : N/A
File # : Bed # : Cash Receipt # : 1937183 Requested By : OPD

Test	Result	Unit	Reference Values

HAEMATOLOGY

COLL: 14/10/2020 - 11:15 RECD: 14/10/2020 - 11:15
VERIFY: 14/10/2020 - 11:58 VERIFY BY:DR. SADIA IJAZ

Test	Result	Unit	Reference Values
HGB *Heamoglobin*			
HGB Haemoglobin	**10.1** L	g / dL	11.5 - 16.5

Sadia

Dr.Sadia Hameed
MBBS DCP MPhil
(Histopath)

Dr.Arif Hussain
MBBS,MCPS,MPhil
(Haematology)

Dr.Shazia Aslam
MBBS MPhil
(Histopath)

Dr.Sadia Ijaz
MBBS MPhil
(Haematology)

Scanned with CamScanner

LAB REPORT

Name	KINZA SHABBIR	W/O	M USMAN	ACCN NO **20-302-0183**
Sex	Female Age 23 Y	OPD Slip/MR #	2010280775 0120086758	
CNIC	[illegible]	Phone #	[illegible]	
Address	Civil Line House No 642 Muh Tariq Abad Sargodh	Requesting Physician	N/A	
File #	1526868	Bed #		
		Cash Receipt #	1945378	Requested By OPD

HAEMATOLOGY

COLL [illegible] RECD [illegible]
VERIFY [illegible] VERIFY BY DR. SADIA [illegible]

Test		Result	Unit	Reference Values
CBC	*Complete Blood Count*			
WBC	White Blood Cells	9.4	x 10 ^ 9 / L	4.0 - 11.0
RBC	Red Blood Cells	4.90	x 10 ^ 12 / L	3.8 - 5.8
HGB	Haemoglobin	12.7	g / dL	11.5 - 16.5
HCT	Haematocrit	38	%	34.0 - 47.0
MCV	Mean Cell Volume	78	fL	78.0 - 95.0
MCH	Mean Cell Haemoglobin	25.5	pg	24.0 - 32.0
MCHC	Mean Cell Haemoglobin Concentration	33.1	g / dL	31.0 - 35.0
PLT	Platelet Count	182	x 10 ^ 9 / L	150.0 - 450.0
MPV	Mean Platelet Volume	**12.7** H	fL	7.5 - 11.0
RDW	Red Cell Distribution Width	**15.4** H	%	11.0 - 14.0
NEUT	Neutrophil Count	4.50	x 10 ^ 9 / L	1.8 - 7.5
LYMPH	Lymphocyte Count	**4.19** H	x 10 ^ 9 / L	1.5 - 4.0
MONO	Monocyte Count	0.47	x 10 ^ 9 / L	0.2 - 0.8
EOS	Eosinophil Count	0.19	x 10 ^ 9 / L	0.0 - 0.6
BASO	Basophil Count	0.02	x 10 ^ 9 / L	0.0 - 0.1

Dr Sadia Hameed
MBBS DCP MPhil
(Histopath)

Dr Arif Hussain
MBBS,MCPS,MPhil
(Haematology)

Dr Shazia Aslam
MBBS MPhil
(Histopath)

Dr Sadia [illegible]
MBBS MPhil
(Haematology)

L = Low C = Critical ... however error / omission is possible. The test result ... [illegible]

Scanned with CamScanner

[illegible] No [illegible]   Sargodha Road, Faisalabad [illegible]

LAB REPORT

Name: KINZA SHABBIR   W/O   M USMAN

Sex: Female   Age: 23 Y

CNIC: 3840355646460   OPD Slip #: 2010280775   120086756

Phone #: 3039446883

ACCN. NO. **20-302-0183**

Address: Civil Line House No 642 Muh Tariq Abad Sargodh

File #: 1526868   Bed #:   Requesting Physician: N/A

Cash Receipt #: 1945376   Requested By: OPD

SPECIAL CHEMISTRY

COLL: 28/10/2020 - 13:33   RECD: 28/10/2020 - 13:33
VERIFY: 28/10/2020 - 14:47   VERIFY BY: DR. SADIA IJAZ

Test	Result	Unit	Reference Values
TSH	2.54	IU / mL	0.27 - 4.20
Free T3	2.88	pg / mL	2.25 - 4.35 Children and adolescence :4.7 - 6.4
Free T4	1.17	ng / dL	0.93 - 1.7

Note : Test is performed on Roche Cobas 6000 employing Electro chemiluminescence Immunoassay (ECLIA) Technology.

Sadia

Dr.Sadia Hameed
MBBS DCP MPhil
(Histopath)

Dr.Arif Hussain
MBBS,MCPS,MPhil
(Haematology)

Dr.Shazia Aslam
MBBS MPhil
(Histopath)

Dr.Sadia Ijaz
MBBS MPhil
(Haematology)

Legend: H = High   L = Low   C = Critical

[illegible] utmost care using best methods / technologies however error / omission is possible. The test should not [illegible] might have been used. This sample is taken with out CNIC confirmation [illegible] signature.

Scanned with CamScanner

[illegible] Sargodha Road, Faisalabad [illegible] 041-8869861 Ext.116 , 172

LAB REPORT

Name : ALLAH MAFI W/O M SAJID AMIN

Sex : Female Age : 33 Y OPD Slip/MR # : 2010290559 0120087110

CNIC. 6110114467684 Phone # : 3454912171

Address: Po Mamokangan Chak No 514 G B Chak Tehsil Ta Requesting Physician : N/A

File # : 1526905 Bed # : Cash Receipt # : 1945862 Requested By : OPD

ACCN. NO.

20-303-0157

HAEMATOLOGY

CBC Complete Blood Count

COLL: 29/10/2020 - 14:05 RECD: 29/10/2020 - 14:05
VERIFY: 29/10/2020 - 14:26 VERIFY BY: DR. SADIA IJAZ

Test	Result	Unit	Reference Values
WBC White Blood Cells	**14.6H**	x 10 ^ 9 / L	4.0 - 11.0
RBC Red Blood Cells	5.27	x 10 ^ 12 / L	3.8 - 5.8
HGB Haemoglobin	14.7	g / dL	11.5 - 16.5
HCT Haematocrit	43	%	34.0 - 47.0
MCV Mean Cell Volume	82	fL	75.0 - 95.0
MCH Mean Cell Haemoglobin	27.9	pg	24.0 - 32.0
MCHC Mean Cell Haemoglobin Concentration	34.1	g / dL	31.0 - 35.0
PLT Platelet Count	214	x 10 ^ 9 / L	150.0 - 450.0
MPV Mean Platelet Volume	10.6	fL	7.8 - 11.0
RDW Red Cell Distribution Width	**15.2H**	%	11.0 - 14.0
NEUT Neutrophil Count	**9.94H**	x 10 ^ 9 / L	1.8 - 7.5
LYMPH Lymphocyte Count	3.65	x 10 ^ 9 / L	1.5 - 4.0
MONO Monocyte Count	**0.96H**	x 10 ^ 9 / L	0.2 - 0.8
EOS Eosinophil Count	0.05	x 10 ^ 9 / L	0.0 - 0.6
BASO Basophil Count	0.03	x 10 ^ 9 / L	0.0 - 0.1

Dr Sadia Hameed
MBBS DCP MPhil
(Histopath)

Dr Arif Hussain
MBBS MCPS MPhil
(Haematology)

Dr Shazia Aslam
MBBS MPhil
(Histopath)

Dr Sadia Ijaz
MBBS MPhil
[illegible]

Scanned with CamScanner

LAB REPORT

Name: ALLAH MAFI W/O M SAJID AMIN

Sex: Female Age :33 Y OPD Slip/MR # :2010290559 0120087110

CNIC: 6110114467684 Phone # : 3454912171

ACCN. NO. **20-303-0155**

Address: Po Mamokangan Chak No 514 G B Chak Tehsil Ta Requesting Physician N/A

File # : 1526905 Bed # : Cash Receipt # : 1945863 Requested By : OPD

Test	Result	Unit	Reference Value

CLINICAL CHEMISTRY

COLL: 29/10/2020 - 14:04 RECD: 29/10/2020 -
VERIFY: 29/10/2020 - 14:47 VERIFY BY:DR. SADIA I

M *Diabetic Monitoring*

Test	Result	Unit	Reference Value
RBS Blood Sugar Random	76.00 L	mg / dL	80.0 - 1[illegible]

Dr.Shazia Aslam
[illegible] MPhil

Dr.Sadia I[illegible]
MBBS M[illegible]
(Haemat[illegible]

Scanned with CamScanner

AL-NOOR LAB & DIAGNOSTIC CENTER

Abubakar Siddique Plaza,Ghalla Mandi Road,Mcclod Gunj, Bahawalnagar

Tel:063-2760333, 0334-4105090 Email: alnoorlabmg@gmail.com

Patient Name	: SALAM ANWAR	Specimen	: Taken in Lab
Father/Husband	:	Reg Date	: Jan 12 2021 04:40 PM
Age/Gender	: 24 Year(s)/Male	Print Date	: Jan 13 2021 11:54 AM
CNIC	:	Reg. Location	: Main Lab Mcclod Gunj
Address	:	Repri. Location	: Main Lab Mcclod Gunj
Consultant	: C/O NOMAN RHC	Contact	: 03006532666
		Lab ID	: 21-292

GLUCOSE PROFILE

Test Name	Patient Value	Reference Range	Units	Remarks
Blood Glucose (Random)	80	60 - 160	mg/dL	

URIC ACID REPORT

Test Name	Patient Value	Reference Range	Units	Remarks
Serum Uric Acid	4.8	2.5 - 7.0	mg/dL	

HEMATOLOGY REPORT

Test Name	Patient Value	Reference Range	Units	Remarks
ESR	5	Upto 9	mm/h	

BLOOD COMPLETE PICTURE

Test Name	Patient Value	Reference Range	Units	Remarks
WBC(TLC)	10.9	3.5 - 10.0	x10³/L	↑
Lymphocytes %	31.7	15.0 - 50.0	%	
MXD % (Mono+Eosin)	5.9	2.0 - 15.0	%	
Neutrophils %	62.4	35.0 - 80.0	%	
Haemoglobin	13.5	14.0 - 17.0	g/dL	↓
MCH	28.1	25.0 - 35.0	Pg	
MCHC	36.5	31.0 - 38.0	g/dL	
RBC	5.16	4.5 - 6.3	x 10¹²/L	
MCV	75.0	75.0 - 100.0	fL	
PCV (HCT)	38.7	35.0 - 55.0	fL	
RDW- SD	44.0	35.0 - 56.0	fl	
RDW-CV	11.2	11.5 - 14.5	%	↓
Platelet Count	268	150 - 400	x10⁹/L	
MPV	7.2	6.5 - 11.0	fl	
PDW	10.7	8.0 - 18.0	fl	
PCT	0.19	0.01 - 9.99	%	
P-LCR	11.5	0.1 - 99.9	%	

Please Note:The test is performed on the state-of-the-art Haematology ANALYZER M-32, fully automated system from MEDONIC, SWITZERLAND.

Not Valid For Any Hon'able Court

Note Electronically Verified Report No Signature Required

ZAFAR IQBAL NASIR
Bsc(Hons),MLT
Medical Technologist

ASAD IMTIAZ BODLA
(DMLT PMF, PB)
Medical Laboratory Technology
Punjab Medical Faculty Lahore

Note:All tests are Performed on the most advance, highly spophistecated, appropiate and state of the art instruments with highly sensitive chemicals under strict conditions and with all care and diligence. However the above Results are NOT the DIAGNOSIS and should be correlated with clinical findings, patient's history, signs and symptoms and other diagnostic tests. Lab to lab variation may occurr. This document is NEVER challengeable at any PLACE/COURT and in any condition.

Scanned with CamScanner

Amina Lab

Name :	Nafeesa	Collection Date :	19.10.2020
Age :	22 Years	Reporting Date :	20.10.2020
Sex :	Female	Ref by :	Self

CBC,s Report

Not Valid For Court

Tests	Result	Unit	Normal Range
Haemoglobin	11.6	g / dl	F : 12.0 - 15.0<br>M:13.0 - 17.0
TLC	5,000	/cmm	4,000 - 11,000
**DLC**			
Neutrophils	48	%	40 - 66
Lymphocytes	49	%	20 - 35
Moncytes	2	%	01 - 03
Eosinophils	1	%	02 - 05
Basophils	0	%	00 - 01
RBC	3.8	Million / cmm	4.1 - 5.9
PCV	35.4	u/l	36 - 48
MCV	92.4	fl/red cell	76 - 96
MCH	30.3	pg/reed cell	28 - 32
MCHC	32.8	g/dl R.B.C	32 - 34
**Platelets**	219	cmm	150 - 450

Lab Incharge

OPP. JAMIA MASJID UMER-E-FAROOQ, NEAR FATEH GARAH AGENCY SIALKOT
CELL: 0333-4662288, 0333-8606563

Scanned with CamScanner

Amina Lab

Name :	Nafeesa	Collection Date :	19.10.2020
Age :	22 Years	Reporting Date :	20.10.2020
Sex :	Female	Ref by :	Self

Laboratory Report

Not Valid For Court

Uric Acid	3.8	mg/dl	Male : 3.5 _ 7.0<br>Female : 2.4 - 5.7
Blood Sugar<br>Random	112	mg/dl	Upto 180 mg/dl

Lab Incharge

OPP. JAMIA MASJID UMER-E-FAROOQ, NEAR FATEH GARAH AGENCY SIALKOT
CELL: 0333-4662288, 0333-8606563

Scanned with CamScanner
