# Supplementary material for: Epidemiological studies of sleep disorder in educational community of Pakistani population, its major risk factors and associated diseases
Source: PLoS One. 2022 Apr 21;17(4):e0266739. doi: 10.1371/journal.pone.0266739 (PMC9022811; doi:10.1371/journal.pone.0266739)
Supplement: S4 File — (PDF) [file pone.0266739.s004.pdf]

Ref #: UO/DoZ/2020/mic

Date: 22-10-2020

BIOETHICAL CLEARANCE CERTIFICATE

**Title of Research:**	Epidemiologic studies of sleep disorder in Educational Community of Pakistani population, its major risk factors and associated diseases
**Department**	Department of Zoology
**Corresponding**	Dr. Muhammad Saleem Khan
**Author**	Ali Umar
**Nature of Work**	Population based survey (Acquisition and analysis of data)

Remarks

It is certified that this research does not involve any hazardous experimental work. The research will be accomplished following the standard. The research has no obvious impact on environment, humans, animals and plant life.

Chairperson Ethical Committee
Dr. Muhammad Wajid
University of Okara

DEPARTMENT OF ZOOLOGY

UNIVERSITY OF OKARA

A Public Sector University Established Under Government of The Punjab Act XIII of 2016

Ref #: UO/D02/2020/mic

Date: 22-10-2020

Verbally consent to serve as subject (participant) in this research entitled "**Epidemiological Studies of Sleep Disorder in Educational Community of Pakistani Population, its Major Risk Factors and Associated Diseases**" was taken from volunteer participants. The nature and general purpose of the research procedure and the known risks involved in this study have been explained to participants. It was decided that information and samples were taken only from those who were willing to participate.

The decision was put in front of Ethics Committee and committee agreed to the decision.

Chairperson Ethics Committee

Dr. Muhammad Saleem Khan
Assistant Professor Zoology
University of Okara

2-KM, Main Multan Road, Renala Khurd, By Pass Okara - Pakistan.
Ph: 044-2635263, 0333-7466063, 0302-6066063 Email: drwajid68@yahoo.com, drwajid68@uo.edu.pk
